# Past and current status of adolescents living with HIV in South Africa, 2005–2017

**DOI:** 10.1186/s13104-022-06006-2

**Published:** 2022-04-09

**Authors:** Inbarani Naidoo, Sinovuyo Takatshana, Ronel Sewpaul, Sean Jooste, Zhou Siyanai, Goitseone Maseko, Sizulu Moyo, Khangelani Zuma, Musawenkosi Mabaso, Zungu Nompumelelo

**Affiliations:** 1 Centre for Community Based Research, Human and Social Capabilities Division, Human Sciences Research Council, Durban, South Africa; 2 Health and Well-Being, Human and Social Capabilities Division, Human Sciences Research Council, Pretoria, South Africa; 3 Health and Well-Being, Human and Social Capabilities Division, Human Sciences Research Council, Cape Town, South Africa; 4 AIDS and Society Research Unit, Centre for Social Science Research, University of Cape Town, Cape Town, South Africa; 5 School of Public Health, University of the Witwatersrand, Johannesburg, South Africa; 6 Health and Well-Being, Human and Social Capabilities Division, Human Sciences Research Council, Durban, South Africa; 7 Department of Psychology, University of Pretoria, Pretoria, South Africa; 8 School of Public Health, University of Cape Town, Cape Town, South Africa

**Keywords:** Adolescents, HIV, Gender, Perinatal, UNAIDS (90-90-90) targets, South Africa

## Abstract

**Objectives:**

This paper reports HIV prevalence, incidence, progress towards the UNAIDS (90-90-90) targets, and HIV drug resistance among adolescents living with HIV in South Africa. We conducted secondary analyses using data extracted from the South African national HIV prevalence surveys (2005–2017). Analyses were stratified by sex and age (10–14 and 15–19-years), presenting weighted descriptive statistics, and realised totals.

**Results:**

HIV prevalence increased from 3.0% in 2012 to 3.7% in 2017, translating to 360 582 (95% CI 302 021-419 144) HIV positive adolescents in 2017. Female adolescents bear a disproportionate HIV burden of 5.6% prevalence versus 0.7% for males. HIV incidence remained relatively stable. For the UNAIDS 90-90-90 targets, approximately 62.3% of adolescents knew their HIV status, 65.4% of whom were on antiretroviral therapy, and of these 78.1% on antiretroviral therapy had attained viral load suppression. There are knowledge gaps pertaining to the magnitude of perinatal infections and postnatal infections, and socio-behavioural risk factors for HIV transmission among adolescents in South Africa. There is still a need for focussed interventions targeting adolescent (1) gender disparities in HIV risk (2) screening for HIV, (3) sustained access and adherence to antiretroviral therapy and (3) retention in care to maintain viral load suppression.

## Introduction

In 2020 approximately 1.7 million children under 15 years were living with HIV (ALHIV) globally; majority of whom reside in sub-Saharan Africa [1]. Despite achievements in HIV containment strategies, South Africa, has the largest population of people living with HIV (PLHIV) estimated at 7.9 million, with adolescents most at risk [2, 3]. Adolescent HIV-related mortality remains high [4, 5]. Hence, prevention and early diagnosis of HIV infection among adolescents are critical [6–8]. HIV infection in adolescents can be acquired postnatally and perinatally, the former due to documented structural and behavioral factors and the latter due to the differential coverage and uptake of mother-to-child transmission programmes [9–11].

Improved understanding of the circumstances of ALHIV is critical in designing adolescent-friendly health services to facilitate their successful transition into adult care. Compared to adults, adolescents have lower rates of HIV testing, disclosure [12], treatment adherence [13] long-term immunologic recovery [14] and viral suppression [15, 16]. In low- and middle-income countries there is limited information available for adolescents at a population level to inform national HIV strategic plans and programming [17].

In South Africa, routine data on ALHIV’s health are also not readily available [3]. Pertinent information on ALHIV can be sourced from a series of national population-based household surveys conducted since 2002 [2, 18–20]. Consequently, we undertook a formative study which was part of a commissioned project entitled “Being ALHIV:’ What do we know about Adolescents Living with HIV in South Africa? In this paper, we report key findings from quantitative analyses [21], focusing on HIV prevalence, incidence, and progress towards the UNAIDS 90–90-90 goals and HIV drug resistance (HIVDR).

## Main text

### Methods

We used data from four South African National HIV Prevalence, Incidence, Behaviour and Communication Surveys (known as “SABSSM”) from 2005 to 2017. These are population-based household surveys, using a multi-stage stratified cluster random sampling design, described in detail in the survey reports [2, 18–20]. Socio-demographic variables were age categories (10–14/12–14/15–19/10–19 years), sex (male/female), population group (Black African, White, Coloured, Indian/Asian), marital status (married/unmarried), currently attending school, employment status, locality type (urban, rural informal/tribal, rural formal/farm) and province. Population group and locality type were described per South Africa’s national census categories [22].

The survey reports describe HIV testing assays for each SABSSM survey [2, 18–20]. HIV prevalence was measured as the percentage of people tested and found to be living with HIV out of the target age group. HIV incidence was defined as the proportion of new infections acquired within the previous 12 months. Incidence data were only available for 2012 and 2017. The incidence estimation was based on a multi-assay algorithm incorporating the Limiting-Antigen Avidity assay (Maxim Bio-medical, Rockville, USA), ART/ARV exposure and HIV viral load. Exposure to ARVs in the 2012 and 2017 surveys was measured by a qualitative determination of the presence of one or more ARVs in the testing panel, using a validated in-house method based on high performance liquid chromatography with tandem mass spectrometry [2, 20]. The testing panel accounted for ARVs used in first, second and third-line ART regimens in the country’s public health HIV programme at the time of each survey [2, 20]. VLS was defined as a viral load of < 1000 copies of HIV RNA/ml. VLS is a measure of ART efficacy and is a proxy for ART adherence and HIV transmission risk [23].

Non VLS (VL ≥ 1000 copies/ml) samples were evaluated for HIVDR using Next Generation Sequencing [2, 24, 25]. Amplification of a 1,084 base pair PCR fragment was performed as described [24]. PCR products were sequenced on the Illumina MiSeq using MiSeq Reagent Kit v3 (Illumina Inc San Diego, CA, USA). Analyses of drug resistance mutations (DRMs) was based on the Stanford v8.0 algorithm, with a 10% prevalence detection threshold [26]. We report on resistance detected overall and by drug class.

Awareness of HIV status was used to estimate.the 90-90-90 UNAIDS’ targets, which stipulates by 2020, 90% of PLHIV know their HIV status, 90% of those diagnosed with HIV receive ART, and 90% of all people receiving ART achieved VLS. The gaps in achieving the UNAIDS’ targets were calculated based on the 90%-81%-73% cascade [27].

#### Statistical analyses

We used cross-sectional benchmarked weights per each survey wave and did not pool these for analyses. We calculated descriptive statistics for the sample characteristics by each indicator variable, using Pearson’s Chi-square tests to detect differences among categorical variables, reporting 95% confidence intervals (CIs). Where CIs did not overlap, this was used to conclude statistical significance. Significance at p ≤ 0.05 are reported. All analyses except incidence estimates were performed in Stata version 15.0 (Stata Corp, College Station, Texas, USA), accounting for the complex survey design. The computational tools for the incidence estimates were developed by the South African Centre for Epidemiological Modelling and Analysis [28].

### Results

#### Socio-demographic characteristics, South Africa 2017

Among 8 762 adolescents tested for HIV in 2017, the majority were Black African (95.4%), attending school (79.1%) and unemployed (99.3%) (Table 1). Over half (53.2%) resided in urban areas, and were mostly from KwaZulu-Natal (27.0%), Gauteng (18.5%), and Mpumalanga (17.5%) provinces.

**Table 1:** Socio-demographic characteristics of adolescents (aged 10–19 years*) living with HIV (aged 10–19 years), South Africa 2017

Variables	N	%
Population group		
Black African	394	95.4
White	4	3.4
Coloured	9	1.2
Indian/Asian	0	0
School attendance*		
Yes	133	79.1
No	30	20.9
Employment status**		
Unemployed	232	99.3
Employed	6	0.7
Locality type		
Urban	148	53.2
Rural informal (tribal areas)	210	42.5
Rural formal (farms)	49	4.3
Province		
KwaZulu-Natal	180	27.0
Gauteng	36	18.5
Eastern Cape	27	12.2
Mpumalanga	92	17.5
Limpopo	16	6.3
North-West	20	5.9
Western Cape	11	7.0
Free State	17	5.1
Northern Cape	8	0.6

^*^Age (weighted): mean (14 years), interquartile range (12–17 years)

^**^Among adolescents living with HIV aged 15–19 years

#### HIV prevalence among adolescents, South Africa 2005–2017

**Fig. 1:**
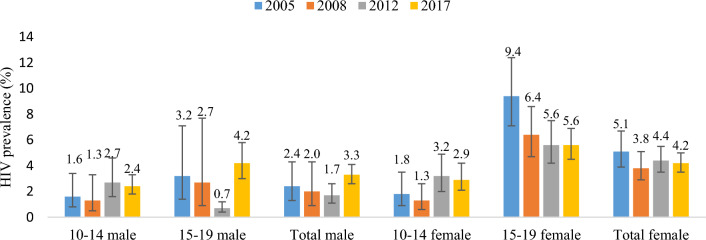
Trends in HIV prevalence among adolescents aged 10–19 years, South Africa 2005, 2008, 2017

Total HIV prevalence declined from 3.6% (95% CI 2.8–4.7) in 2005 to 3.0% (95% CI 2.5–3.7) in 2012, then increased to 3.7% in 2017 (95% CI 3.2–4.3) (Fig. 1). Despite fluctuations over time, females carried the highest burden, with their HIV prevalence ranging from 3.8% to 5.1% compared to 2.0% and 3.3% in males between 2005 and 2017. In 2012, HIV prevalence among female adolescents aged 15–19 years was significantly higher than males—5.6% (95% CI 4.2–7.5) vs 0.7% (95% CI 0.4–1.2).

The 3.7% prevalence in 2017 translates to 360,582 ALHIV (95% CI 302,021–419,144), comprising 136,913 aged 10–14 years and 223,669 aged 15–19 years. Of these, 202,923 were female, and 157,659 were male. In 2017, HIV prevalence among female adolescents aged 15–19 years remained unchanged at 5.6% (95% CI 4.5–6.9) whilst for their male counterparts, it increased to 4.2% (95% CI 3.0–5.8) from 0.7% in 2012.

#### HIV disclosure among ALHIV aged 15–19 years, 2017

Among ALHIV who knew their status, more females, (77.6%, 95% CI 58.6–89.4), said they had disclosed their HIV status to a main sexual partner compared to males (11.5%, 95% CI 2.4–40.8), although there was a small sample for males (n = 9).

#### HIV incidence estimates for ALHIV, 2012 and 2017

HIV incidence among adolescents aged 10–19 years was 0.50% (95% CI 0.44–0.56) in 2012, declining to 0.39% (95% CI 0.35–0.43) in 2017. HIV incidence in adolescents aged 15–19 years was 0.87% (95% CI 0.82–0.92) in 2012 and remained relatively stable at 0.82% (95% CI 0.74–0.90) in 2017. There were limited data to estimate HIV incidence in adolescents aged 10–14 years [2] and to disaggregate HIV incidence by sex in these age groups.

#### VLS and HIVDR among ALHIV, 2017

HIV viral load results were available for 398 10–19 years ALHIV. Among these, 48.9% (95% CI 41.5–56.3) had VLS (VL < 1000 copies/ml). A higher proportion (54%, 95% CI 41.9–65.7) of ALHIV aged 10–14 years had VLS compared to their older counterparts (45.7%, 95% CI 36.5–55.3), but these differences were non significant.

Of n = 182 ALHIV samples not virally suppressed, n = 106 were successfully amplified and sequenced and of these, 33.7% (95% CI 22.8–46.7, n = 33) had HIVDR. Of the 33 samples, 64.5% (95% CI 43.5–81.1, n = 18) had non-nucleoside reverse transcriptase inhibitor (NNRTI) mono resistance, while 30.4% (95% CI 15.2–51.6, n = 13) had dual NNRTI and nucleoside reverse transcriptase inhibitor resistance. We found no mutations to protease inhibitor drugs. Of the samples successfully amplified and sequenced for DRMs and analysed for ART exposure (n = 82), 12% (95% CI 6.0–22.5) were exposed to ART).

#### Progress towards UNAIDS 90-90-90 targets for adolescents, South Africa 2017

Approximately 62.3% (95% CI 54.7–69.2) of ALHIV aged 10–19 years knew their HIV status and of these 65.4% (95% CI 54.3–75.0) were on ART. Among ALHIV on treatment 78.1% (95% CI 69.0–85.1) were virally suppressed.

Overall awareness of their HIV status was significantly lower (p < 0.001) for ALHIV aged 10–14 years at 44.5% (95% CI 33.1–56.5) compared to 73.1% for those aged 15–19 years (95% CI 64.2–80.5). Among ALHIV who knew their status, ART uptake was significantly higher (p = 0.001) among 10–14-year-old adolescents (89.9%, 95% CI 72.9–96.7) compared to their 15–19 -year-old counterparts (57.9%, 95% CI 45.6–69.3). For ALHIV who were on treatment, 75.2% (95% CI 55.2–88.1) of those aged 10–14 years had VLS, and a similar proportion of 15–19-year-old ALHIV had VLS (79.5%, 95% CI 68.5–87.4).

**Fig. 2:**
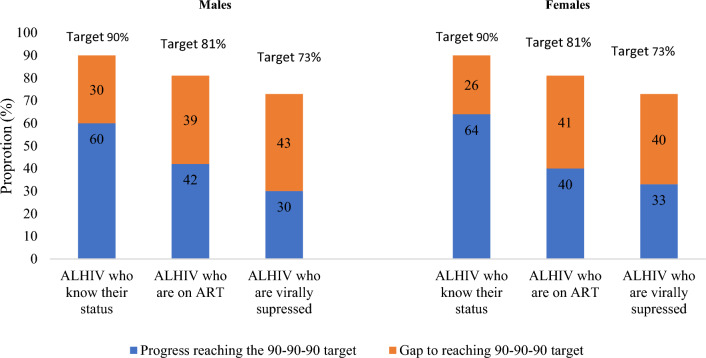
Gaps in achieving UNAIDS targets among adolescents living with HIV, South Africa 2017 based on the 90–81–73% cascade

Similar proportions of male and female ALHIV aged 10–19 years knew their HIV status (1st 90) i.e., 60.1% (95% CI 49.6–69.7) for males and 63.9% for females (95% CI 53.4–73.3). However, ART uptake was lower among females at 62.1% (95% CI 50.1–72.8) than males (70.5%, 95% CI 50.7–84.7) (2nd 90). Among ALHIV on ART, more females had VLS at 82.8% (95% CI 71.3–90.3) than their male counterparts (71.7%, 95% CI 55.4–83.9) (3rd 90). Overall, the gap in attaining VLS was 43% among male ALHIV and 40% among female ALHIV (Fig. 2).

### Discussion

There were approximately 360 600 ALHIV aged 10–19 years in South Africa in 2017. Female adolescents bear a disproportionate burden of HIV compared to their male counterparts. The gender differential is consistent with HIV incidence among adolescents living in sub-Saharan Africa [29] and can be attributed to socio-cultural practices. These include early sexual debut, having multiple sexual partners, age disparate relationships, which co-exist with economic disparities and gender based power dynamics including gender-based violence [21, 30]. HIV incidence was largely unchanged in the 15–19 year age group between 2012 and 2017, possibly attributable to interventions targeting adolescent girls and young women. Although young people share concerns and needs as adolescents, HIV programmes must recognize their heterogeneity. Special attention must be given to female ALHIV needs, both in prevention and care programmes.

Progress towards the UNAIDS target of the first 90 at 62.3% being aware of their HIV status, is substantially lower compared to the general population, where 85% of PLHIV aged 15–64 years knew their status [2]. The reasons for younger adolescents not knowing their status are multi-faceted. For instance, they may have not been informed by a parent/caregiver, nor have a reason to test if they were not engaged in sexual activity, or if they are in good health. This can be partly attributed to complexities of parental disclosure due to fear and social stigma, perceived child's negative emotional reaction and concerns about the child being too young and immature to understand a diagnosis of being HIV positive [31].

We found low levels of ART use and low levels of VLS among ALHIV. Like other African region-based studies, ART uptake was lower for females than males [32, 33]. There might be several reasons for a gender differential in ART initiation, including female children being less likely to be taken for health care and later ART initiation in females, compared to males [32]. Lastly, this study is among the first to estimate levels of HIVDR in ALHIV in a household survey in South Africa [2, 25]. Continued monitoring of HIVDR in ALHIV and in the general population is needed, particularly to detect pre-treatment HIVDR [34, 35].

### Conclusion

The high HIV prevalence among female adolescents and steady incidence highlight the need to prevent new infections among adolescents. There remains a need for improved access to quality sexual and reproductive health services that are youth-friendly and gender sensitive.

### Limitations

This study is limited by the small sample size, particularly for those aged 10–14 years. Collection of blood specimens for determination of HIV status in this group are influenced by parents’ or caregivers’ reluctance to give consent as mentioned by other researchers in this area [36]. The samples used to estimate incidence for this age group were small. Therefore, these estimates should be interpreted with caution. Nevertheless, this study contributes to the paucity of information available about ALHIV in South Africa. The 2017 wave is the most recent cross sectional, national HIV prevalence survey for South Africa, and the 6th wave is currently underway.

## Data Availability

The datasets used are available through the Human Sciences Research Council data research repository via the following link: http://curation.hsrc.ac.za/Datasets-PFAJLA.phtml.

## Abbreviations

ALHIV: Adolescents living with HIV
ART/ARV: Antiretroviral therapy
NNRTI: Non-nucleoside reverse transcriptase inhibitor
PLHIV: Persons living with HIV
SABSSM: South African National HIV Prevalence, Incidence, Behaviour and Communication Surveys
VLS: Viral load suppression
HIVDR: HIV drug resistance
