# Correction: Past and current status of adolescents living with HIV in South Africa, 2005–2017

**DOI:** 10.1186/s13104-024-06730-x

**Published:** 2024-03-12

**Authors:** Inbarani Naidoo, Sinovuyo Takatshana, Ronel Sewpaul, Sean Jooste, Zhou Siyanai, Goitseone Maseko, Sizulu Moyo, Khangelani Zuma, Musawenkosi Mabaso, Zungu Nompumelelo

**Affiliations:** 1 Centre for Community Based Research, Human and Social Capabilities Division, Human Sciences Research Council, Durban, South Africa; 2 Health and Well-Being, Human and Social Capabilities Division, Human Sciences Research Council, Pretoria, South Africa; 3 Health and Well-Being, Human and Social Capabilities Division, Human Sciences Research Council, Cape Town, South Africa; 4 AIDS and Society Research Unit, Centre for Social Science Research, University of Cape Town, Cape Town, South Africa; 5 School of Public Health, University of Cape Town, Cape Town, South Africa; 6 School of Public Health, University of the Witwatersrand, Johannesburg, South Africa; 7 Health and Well-Being, Human and Social Capabilities Division, Human Sciences Research Council, Durban, South Africa; 8 Department of Psychology, University of Pretoria, Pretoria, South Africa

**BMC Research Notes (2022) 15:132**

10.1186/s13104-022-06006-2

Following the publication of the original article, we were notified that the authors’ given and family names had been interchanged.

The correct authors’ names are shown in the author list of this Correction. The original article has been revised.

